# Validation of Perioperative Troponin Levels for Predicting Postoperative Mortality and Long-Term Survival in Patients Undergoing Surgery for Hepatobiliary and Pancreatic Cancer

**DOI:** 10.3390/jcdd11040130

**Published:** 2024-04-22

**Authors:** Dimitrios E. Magouliotis, Evangelos Tatsios, Grigorios Giamouzis, Athina A. Samara, Andrew Xanthopoulos, Alexandros Briasoulis, John Skoularigis, Thanos Athanasiou, Metaxia Bareka, Christos Kourek, Dimitris Zacharoulis

**Affiliations:** 1 Unit of Quality Improvement, Department of Cardiothoracic Surgery, University of Thessaly, Biopolis, 41110 Larissa, Greece; 2 Department of Surgery, University of Thessaly, Biopolis, 41110 Larissa, Greece; evangelos.tats@gmail.com (E.T.); at.samara93@gmail.com (A.A.S.); zacharoulis@uth.gr (D.Z.); 3 Department of Cardiology, University of Thessaly, Biopolis, 41110 Larissa, Greece; grgiamouzis@gmail.com (G.G.); andrewvxanth@gmail.com (A.X.); iskoular@gmail.com (J.S.); 4 Department of Therapeutics, Faculty of Medicine, National and Kapodistrian University of Athens, 11528 Athens, Greece; alexbriasoulis@gmail.com; 5 Department of Surgery and Cancer, Imperial College London, St Mary’s Hospital, London W2 1NY, UK; t.athanasiou@imperial.ac.uk; 6 Department of Anesthesiology, University of Thessaly, Biopolis, 41110 Larissa, Greece; barekametaxia@hotmail.com; 7 Department of Cardiology, 417 Army Share Fund Hospital of Athens (NIMTS), 11521 Athens, Greece; chris.kourek.92@gmail.com

**Keywords:** troponin, TnT, pancreatic cancer, HPB, hepatobiliary cancer, MINS

## Abstract

**Background:** Hepatopancreato and biliary (HPB) tumors represent some of the leading cancer-related causes of death worldwide, with the majority of patients undergoing surgery in the context of a multimodal treatment strategy. Consequently, the implementation of an accurate risk stratification tool is crucial to facilitate informed consent, along with clinical decision making, and to compare surgical outcomes among different healthcare providers for either service evaluation or clinical audit. Perioperative troponin levels have been proposed as a feasible and easy-to-use tool in order to evaluate the risk of postoperative myocardial injury and 30-day mortality. The purpose of the present study is to validate the perioperative troponin levels as a prognostic factor regarding postoperative myocardial injury and 30-day mortality in Greek adult patients undergoing HPB surgery. **Method:** In total, 195 patients undergoing surgery performed by a single surgical team in a single tertiary hospital (2020–2022) were included. Perioperative levels of troponin before surgery and at 24 and 48 h postoperatively were assessed. Model accuracy was assessed by observed-to-expected (O:E) ratios, and area under the receiver operating characteristic curve (AUC). Survival at one year postoperatively was compared between patients with high and normal TnT levels at 24 h postoperatively. **Results:** Thirteen patients (6.6%) died within 30 days of surgery. TnT levels at 24 h postoperatively were associated with excellent discrimination and provided the best-performing calibration. Patients with normal TnT levels at 24 h postoperatively were associated with higher long-term survival compared to those with high TnT levels. **Conclusions:** TnT at 24 h postoperatively is an efficient risk assessment tool that should be implemented in the perioperative pathway of patients undergoing surgery for HPB cancer.

## 1. Introduction

Hepatopancreato and biliary (HPB) tumors represent some of the leading cancer-related causes of death worldwide and the fourth cause of cancer mortality in the US alone [1], with a significant proportion of patients undergoing potentially curative surgery. Generally, these tumors are associated with a poor prognosis, given that many types are diagnosed at a late stage [1]. In fact, tumors like pancreatic adenocarcinoma located at the tail of the pancreas might progress in a silent, subclinical pattern without giving any symptoms, thus making early diagnosis and curative treatment difficult. In this context, the accurate evaluation of the perioperative risk is crucial to facilitate the shared decision making (SDM) and informed consent processes among physicians, surgeons, and patients undergoing surgery and to enhance clinical practice during the perioperative pathway. Furthermore, the implementation of an accurate risk stratification tool enables the actual comparison of surgical outcomes among different healthcare providers for either service evaluation or clinical audit, thus enabling the design and implementation of quality improvement initiatives.

Over the last few decades, several risk stratification tools have been introduced into clinical practice [2]. In fact, risk stratification tools may be subdivided into risk scores and risk prediction models. Both are usually developed using multivariable analysis of risk factors targeting a specific outcome [2]. When discussing risk assessment tools in HPB surgery, it is essential to consider the sensitivity and specificity of the American College of Surgeons National Surgical Quality Improvement Program (ACS-NSQIP), the Revised Cardiac Risk Index (RCRI), and the Physiological and Operative Severity Scoring for the Enumeration of Mortality and Morbidity (POSSUM). These are widely used tools in surgical risk assessment, but their effectiveness in HPB surgery requires careful evaluation [3]. The ACS-NSQIP is known for its comprehensive data collection and risk-adjusted surgical outcomes, but its sensitivity and specificity in predicting complications specifically in HPB surgery need to be studied further. Similarly, the RCRI and POSSUM, initially designed to assess cardiac risk in noncardiac surgery, may have limitations when applied to HPB procedures due to the unique nature of these surgeries [4,5]. When considering the value of preoperative risk assessment tools in HPB surgery, it is also important to address the importance of the Surgical Outcomes Risk Tool in this context [5]. The SORT has been specifically designed to evaluate the risk associated with complex surgical procedures, such as hepatobiliary surgery. The development and inclusion of an HPB-specific prediction platform, similar to SORT, are critical in accurately assessing the risks and predicting outcomes in patients undergoing these intricate surgeries. By incorporating disease process-specific metrics and factors, the SORT can provide a more tailored and accurate risk assessment for HPB surgeries. Furthermore, the incorporation of the SORT in preoperative risk assessment can also have implications on the reimbursement models. It can address the current limitations in available platforms and ensure that the complexity and specific considerations of HPB surgeries are adequately reflected in the assessment, potentially leading to more accurate cost evaluation and resource allocation. According to a recent study from our team [6], the SORT has demonstrated excellent discrimination (area under the curve: 0.98) and calibration traits in predicting 30-day mortality regarding patients undergoing pancreatic surgery.

Despite the increasing interest in more advanced, sensitive, and accurate risk prediction methods, risk stratification models remain the most easily accessible choice for this purpose. Nonetheless, even today, they are not routinely used in everyday clinical practice, possibly due to poor awareness amongst clinicians regarding the available tools, along with certain concerns regarding their complexity and accuracy [5]. Given the high risk of perioperative adverse cardiac events in oncologic surgical patients, the calculation of the perioperative changes in troponin T (TnT) levels has been proposed as a potential prognostic marker for such events, but its implementation in clinical practice has not been widely adopted and investigated [7].

Recently, the diagnosis of myocardial injury in the setting of noncardiac surgery (MINS) was introduced to focus the perioperative attention on the prognostic relevance of ischemic troponin level changes following noncardiac surgery [7]. The diagnostic criteria for MINS are based on measuring peak fourth-generation troponin T (TnT) plasma levels over 0.03 ng/mL, attributed to myocardial ischemia, which occurs within 30 days after noncardiac surgery [7]. Whether or not MINS is associated with adverse events or poor prognosis in HPB surgical patients is not known. Nonetheless, the high intrinsic risk of postoperative complications associated with HPB surgical patients makes them a group of particular clinical interest. Based on the potential significance of TnT as a perioperative prognostic marker, we designed a prospective study aiming to determine (i) the clinical characteristics of HPB surgery patients with and without MINS, (ii) the 30-day outcomes for HPB surgery patients with and without MINS, and (iii) the long-term survival of patients with perioperative TnT elevation. Herein, we present the early outcomes of this study. 

## 2. Materials and Methods

### 2.1. Data Extraction

The present study was conducted under the protocol agreed upon by all authors. This is a prospective cohort study that adheres to the Strengthening the Reporting of Observational Studies in Epidemiology (STROBE) statement [8]. Data were obtained from a prospectively maintained database. We included all consecutive patients undergoing surgery for HPB cancer between 1 January 2020 and 31 August 2022 by the same surgical team led by the senior author (D.Z.) at the Department of Surgery, University Hospital of Larissa, Greece, with complete perioperative and one-year follow-up data available. We excluded all patients who did not provide informed consent and those who did not follow our protocol for measuring TnT levels. Ethical approval was obtained by the Scientific Committee of the University Hospital of Larissa (Protocol Number: 41743/10-01-20). Informed consent was granted from all the included patients and securely stored. No imputation methods were used regarding missing data.

Data on age, gender, body mass index (BMI), previous operations, American Society of Anesthesiology (ASA) grade, operative priority, and type of procedure were prospectively collected. Mortality was defined as any death that occurred during the first 30 days or within the hospital stay if longer than 30 days. We also performed a one-year survival follow-up for all included patients. The predicted risk of mortality was determined using the Surgical Outcome Risk Tool (SORT) [5]. The calculation of the SORT score was performed by employing the method and the web-based calculator developed by Protopappa et al. [5], along with the updated version incorporating subjective information [9]. The SORT model implements the following variables: ASA physical status (PS), operative priority (elective, urgent, and immediate), surgical specialties (gastrointestinal, thoracic, or vascular surgery), surgical severity (major/complex), malignancy status, age (65–79 or ≥80 years), and risk for postoperative pancreatic fistula (POPF) (high or low risk). The diagnostic criteria for MINS comprise a peak fourth-generation troponin T (TnT) plasma level of 0.03 ng/mL or higher judged to be due to myocardial ischemia, which occurs within 30 days after noncardiac surgery [4]. We used the Flex AQT90 Delta Medical^®^ (Delta Medical S.A., Athens, Greece) system to measure the TnT levels. We employed fourth-generation TnT plasma levels instead of fifth-generation TnT levels based on the hospital equipment that was accessible to our institution and across departments to ensure the homogeneity of the data. In addition, fourth-generation TnT measuring is described to predict MINS in the American Heart Association report on perioperative TnT measuring [10]. In this period, troponin T was scheduled to be measured daily at three time points: preoperatively at the admission, at 24, and 48 h postoperatively. The rationale behind the time points selection was based on the observation that MINS typically occurs in the first 72 h postoperatively [10].

### 2.2. Endpoints

The primary endpoint of the study was to assess the discrimination and calibration traits of TnT, measured in three time points (preoperatively, at 24, and 48 h postoperatively), in predicting mortality. A secondary endpoint was to assess the hypothesis of noninferiority in terms of one-year survival of patients with normal TnT levels at 24 h postoperatively compared to those with high levels of TnT at 24 h postoperatively. The cut-off level of TnT was set as 0.03 ng/mL. 

### 2.3. Statistical Analysis

We evaluated the discrimination traits (the ability to separate those patients who did from those who did not die) and calibration traits (the ability to predict mortality rates in agreement with actual observed mortality rates) of preoperative TnT, along with TnT at 24 and 48 h postoperatively. Discrimination was evaluated by producing receiver operating characteristic (ROC) curves and by calculating the area under the ROC curve (AUC). The AUC was determined by calculating the 95% confidence intervals and compared using nonparametric paired tests, as described by DeLong et al. [11]. We defined poor, fair, and excellent model discrimination traits as the AUC levels of <0.70, 0.70–0.79, and 0.80–1.00, respectively [11]. 

The calibration regarding each model was calculated by estimating the predicted mortality (expected) and then compared with the true mortality (observed). The observed/expected ratio of 1 represents perfect accuracy, a ratio <1 indicates overprediction of mortality rate, and a ratio of >1 indicates underestimation. Calibration was further evaluated using the Hosmer–Lemeshow (H-L) goodness-of-fit test, defining a lack of fit as a *p*-value ≤ 0.05 [11]. Cases where the outcome variable separated the predictor variable completely were defined as perfect separation. 

We also compared survival at one year postoperatively between patients with high and normal TnT levels at 24 h postoperatively by constructing a Kaplan–Meier graph. A *p*-value < 0.05 was set as the threshold indicating a statistically important result. Finally, we employed the Mantel–Haenszel statistical method to estimate the hazard ratio (HR) with its 95% confidence intervals (95% CIs). Finally, we performed multiple logistic regression to adjust TnT for age, preoperative hematocrit, and Surgical Outcome Risk Tool (SORT) stratification, which were considered potential confounding variables.

All data were analyzed using Microsoft^®^ Excel 16.61 (Microsoft, Redmond, Washington, DC, USA, 2024) and Prism^®^ GraphPad 10.0.2 for Mac (GraphPad Software, San Diego, CA, USA, 2024).

## 3. Results

### 3.1. Baseline Characteristics

**Figure 1:**
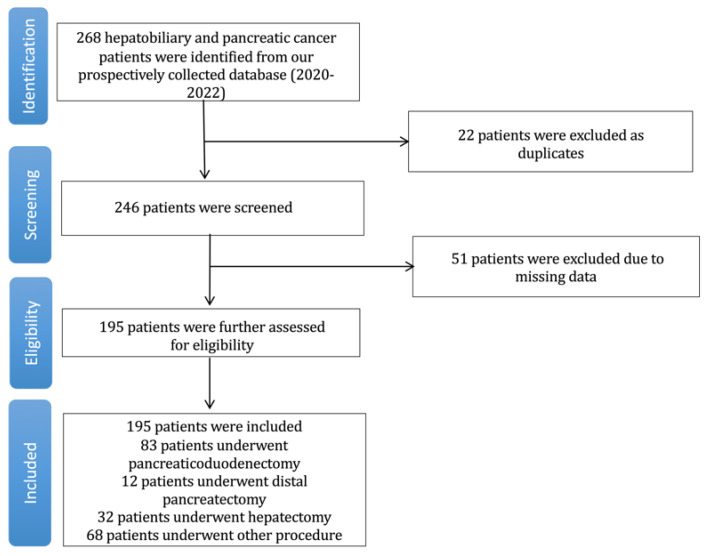
Trial flowchart.

We report our outcomes according to The Strengthening the Reporting of Observational Studies in Epidemiology (STROBE) guidelines [9]. The trial flow regarding the data extraction strategy is presented in Figure 1. A total of 268 patients were screened, and 195 patients were finally included. Patients’ baseline characteristics are presented in Table 1. Overall, 49 (25.1%) patients were females, with a mean age of 64.2 (standard deviation—SD: 11.4) years. The majority of the patients underwent an elective procedure (81.0%). A total of 83 (42.6%) patients underwent pancreaticoduodenectomy, 12 (6.2%) underwent distal pancreatectomy, and 32 (16.4%) underwent hepatectomy. The mean mortality risk according to the SORT was 1.35%. In addition, 13.8% of the included patients presented MINS. The overall 30-day mortality rate was 6.6%. The mean long-term follow-up was 227 ± 131.1 days, with a drop-out rate of 27.6% for the high TnT group and 21.1% for the normal TnT group at 24 h considering the one-year follow-up. 

### 3.2. Validation of Perioperative TnT Levels

**Figure 2:**
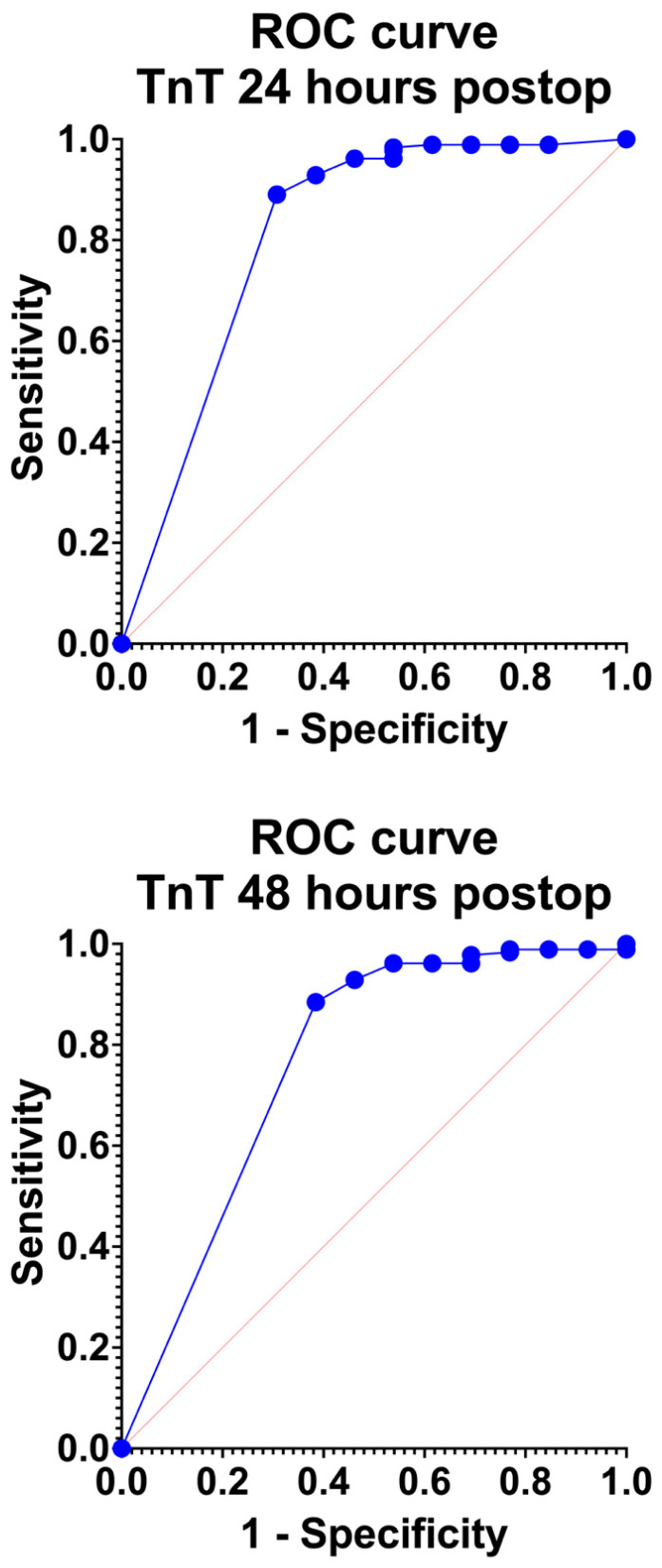
ROC curves regarding the discrimination of troponin T (TnT) levels at 24 and 48 h postoperatively.

As demonstrated in Table 2 and Figure 2, preoperative levels of TnT were associated with poor discrimination [AUC: 0.60 (95% CI: 0.42–0.77); *p* = 0.242]. Nonetheless, the TnT levels at 24 h postoperatively were significantly associated with excellent discrimination traits [AUC: 0.88 (95% CI: 0.82–0.93); *p* < 0.001]. Finally, TnT at 48 h postoperatively was associated with a fair discrimination level [AUC: 0.76 (95% CI: 0.60–0.93); *p* = 0.002]. In comparison, SORT V1 [AUC: 0.75 (95% CI: 0.60–0.90); *p* = 0.003] and POSSUM [AUC: 0.64 (95% CI: 0.49–0.79); *p* = 0.09] were associated with lower discrimination traits in the present analysis. In addition, at all three time points, TnT was associated with low Hosmer–Lemeshow values, thus providing a good-performing calibration. Nonetheless, TnT levels at both 24 and 48 h postoperatively underestimated mortality determined by observed/expected ratios of >1. We also compared pre- and postoperative TnT levels, but no significant difference was found.

### 3.3. Evaluation of TnT Levels in Terms of Long-Term Survival

**Figure 3:**
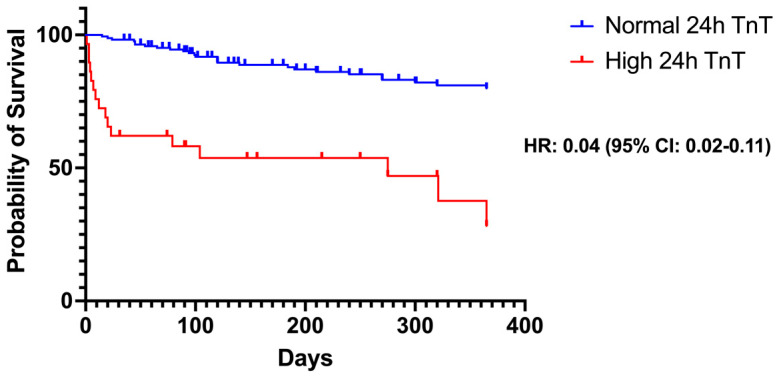
Kaplan–Meier curves regarding the survival at one-year follow-up and the difference between the high troponin T (TnT) and normal TnT groups at 24 h postoperatively.

Patients in the high 24 h postoperatively TnT group demonstrated significantly lower survival at one year postoperatively [AUC: 0.04 (95% CI: 0.02–0.11); *p* < 0.001], as demonstrated in Figure 3. This finding suggests the important role of 24 h postoperative TnT levels on the long-term prognosis of these patients. 

We also adjusted 24 h TnT levels for age, preoperative hematocrit, and SORT stratification. No significant difference in terms of odds ratio was reported, as demonstrated in Table 3. Finally, in Table 4, we present the perioperative characteristics of patients with either normal or high TnT levels preoperatively, at 24 and 48 h postoperatively. 

## 4. Discussion

The current study is the first to evaluate the validity of perioperative TnT levels as prognostic markers for mortality and the long-term survival of patients undergoing hepatobiliary and pancreatic surgery. The outcomes provided by our study have a direct impact on clinical practice, suggesting the potential role of TnT as a prognostic marker that should be measured during the perioperative pathway of patients considered high-risk for presenting MINS. Nonetheless, in the present study, we also evaluated the potential value of perioperative TnT levels in predicting long-term survival. The present study demonstrates the early outcomes of a prospective study we conducted. Nonetheless, they represent a good first line of evidence on the value of perioperative TnT measuring.

Despite the increasing interest in the role of TnT perioperative changes, along with the presentation of MINS, currently, there is still only limited evidence on the long-term impact of perioperative TnT elevation on survival regarding patients undergoing noncardiac surgery [8,10]. Nonetheless, intraoperative factors, such as anesthesia management and surgery-related factors, have been associated with a significant effect on postoperative myocardial injury and death [12,13]. Our findings suggest an increased risk of mortality, which is consistent with the hypothesis of MINS negatively affecting the trajectory of a patient with cancer undergoing surgery, although not all postoperative deaths were attributed to a cardiac event. To our knowledge, no other studies have investigated associations between MINS and long-term oncological outcomes in patients undergoing HPB cancer surgery. On the other hand, there is similar evidence regarding the effect of increased postoperative TnT levels on the long-term survival of patients undergoing colorectal cancer surgery [14]. Clinical physicians typically tend to primarily rely on their clinical experience and guidelines to determine whether to perform presurgical TnT testing, which may include recent myocardial infarction, recent acute myocardial injury, known coronary artery disease, known heart failure, and symptoms of angina. Nonetheless, to harness real-world big data analysis outcomes, where actual data often integrates a multitude of complex situations, and their conclusions more intuitively reflect clinical scenarios and adapt to the complexity of the real world, we need more objective measures than clinicians’ instincts. In this study, it was evident that certain patients who were generally considered to be at low or intermediate risk underwent troponin testing perioperatively and presented MINS. 

Another important finding of our study was the difference in TnT levels at different time points. In fact, TnT at 24 h postoperatively was associated with the best-predicting traits compared to TnT preoperatively and at 48 h postoperatively. This may be related to the etiology of MINS. In fact, the main mechanism of MINS is oxygen supply/demand mismatch ischemia, either related to a coronary or a noncoronary cause [15]. A patient undergoing surgery faces high levels of perioperative stress, thus injuring tissues, which further complicates oxygen supply/demand mismatch ischemia. However, fatal cases of perioperative myocardial ischemia have reportedly been related to acute coronary syndrome with coronary plaque rupture [16,17]. 

In further analysis, we demonstrated that patients with high levels of TnT at 24 h postoperatively were associated with lower long-term survival. This indicates that elevated TnT perioperatively was indeed related to long-term outcomes and suggests that prior studies underestimated the harms and risks derived from elevated TnT levels by only assessing short-term outcomes. These results highlight the fact that the occurrence of MINS should not be ignored, even in younger and fitter patients undergoing noncardiac surgery, and suggest that theoretically “low-risk” patients who are at real risk may drop out from perioperative TnT screening even though they need it. Potentially, there is an interplay between the inflammatory status of oncologic patients and the underlying inflammation of cardiovascular diseases that contributes to a worse long-term outcome. In the same context, in the recent perioperative guidelines, a recommendation has been included for perioperative TnT investigations to screen for MINS, but the details vary among different associations. In fact, the guidelines conducted by the American College of Cardiology/American Heart Association and the European Society of Cardiology/Anesthesiology suggest the routine TnT measurement for patients with ischemic symptoms or those being at high risk for cardiovascular events [18,19]. The more recent Canadian Cardiovascular Society guidelines add a strong recommendation for performing daily TnT measurements for 2–3 days following surgery in patients with a >5% cardiovascular risk, based on the finding that the majority of clinically important MINS cases might stay undetected otherwise [20,21]. Our results provide an additional clue on the role of TnT at 24 h postoperatively, which the current guidelines may also need to implement. Notably, current guidelines are supported by the cost–benefit of perioperative TnT screening [22], so this may also need to be investigated for younger and healthier patients. In the same context, we suggest that oncologic patients with increased TnT levels at 24 h postoperatively should be scheduled for cardiac investigation to be prescribed the appropriate pharmacologic treatment following discharge and should undergo a thorough and close cardiologic follow-up.

A certain limitation of the present study is associated with the study design itself, as it is a single-institution study with a small study sample. As in any observational analysis, unobserved confounding factors might have distorted our results [23]. However, to confront this limitation, we adjusted data for potential cofounders. The cofounders we used were age, preoperative hematocrit, and SORT scores. Another limitation is that censored death data during follow-up may not be random. Multiple data sources need to be adopted to attenuate misclassification bias in the future. In addition, a great range of different surgical operations were included, thus increasing the heterogeneity of the population. To adjust our outcomes regarding this potential source of bias, we performed multivariate analysis using the SORT score as a confounding factor, given that the SORT takes into consideration the type of surgical operation. Finally, both TnT levels at 24 and 48 h underestimated mortality. For this reason, we suggest that this information should be used in conjunction with other well-validated risk stratification tools, while future studies with larger samples should investigate in-depth the most appropriate cut-off value. Nonetheless, the data were prospectively collected, the patients were consecutive, the surgical team was the same, and the surgeon’s bias regarding patient/approach selection was minimized, as this was decided based on MDT suggestions and patients’ choices after extensive counseling. Additionally, the follow-up period was limited, and more correlations between TnT levels and adverse events need to be assessed. However, the present study reflects only the primary outcomes, and these issues will be assessed in the final report. 

Taking everything into consideration, the current outcomes demonstrate that measuring TnT levels at 24 h postoperatively is an easy, feasible, and efficient risk stratification tool that should be implemented in clinical practice for intermediate- and high-risk patients and should be further investigated for younger and healthier patients undergoing HPB oncologic surgery. 

## 5. Conclusions

In the present study, we validated the perioperative levels of TnT as a mortality-prediction and risk stratification tool in adult patients undergoing surgery for HPB cancer. TnT at 24 h postoperatively demonstrated the best-performing discrimination and calibration compared with TnT preoperatively and at 48 h postoperatively. In addition, patients with higher levels of TnT at 24 h demonstrated a lower long-term survival rate compared to those with normal TnT levels. Taking everything into consideration, TnT at 24 h postoperatively is a feasible and efficient risk stratification tool that could be easily implemented in the perioperative pathway of patients with HPB cancer undergoing surgery. 

## Figures and Tables

**Table 1:** Patient baseline characteristics.

Demographics	Number of Patients, n = 195
Female, n (%)	49 (25.1)
Mean age, years (SD)	64.2 (11.4)
Age ≥ 70 (%)	60 (30.8)
BMI, (SD)	26.5 (1.9)
Mean previous operations, n (SD)	1.9 (1)
ASA class, n (%)	
I	38 (19.4)
II	98 (50.3)
III	44 (22.6)
IV	15 (7.7)
SORT (SD)	1.35 (1.9)
Operative priority	
Elective	158 (81.0)
Acute	37 (19.0)
Cancer site, n (%)	
Pancreas	164 (84.1)
Stage	
Resectable	110 (67.1)
Borderline resectable	54 (32.9)
PDAC	139 (84.8)
NET	14 (8.5)
Other	11 (6.7)
Hepatobiliary	31 (15.9)
Stage	
I, II	25 (80.6)
III	6 (19.4)
Neoadjuvant treatment, n (%)	87 (44.6)
Surgical operation, n (%)	
Pancreaticoduodenectomy	83 (42.6)
Distal pancreatectomy	12 (6.2)
Hepatectomy	32 (16.4)
Other procedures	68 (34.8)
Blood loss, n (%)	
<100 mL	61 (31.3)
101–500 mL	92 (47.2)
501–1000 mL	36 (18.5)
>1001 mL	6 (3)
Anesthesia events, n (%)	9 (4.6)
Severity of procedure, n (%)	
Major/Complex	182 (93.3)
30-day mortality	13 (6.6)

Abbreviations: ASA: American Society of Anesthesiologists.

**Table 2:** Discrimination and calibration of troponin (TnT) for predicting mortality in pancreatic cancer patients undergoing surgery.

Predictive Marker	O	E	O:E	Discrimination		Calibration	
				AUC (95% CI)	*p*	H-L	*p*
Preoperative TnT	13	0	-	0.60 (0.42–0.77)	0.242	1	0.32
TnT at 24 h postoperatively	13	5	2.6	0.88 (0.82–0.93)	<0.001	1.38	0.24
TnT at 48 h postoperatively	12	2	6	0.75 (0.57–0.92)	0.004	2.7	0.1

Abbreviations: O: observed; E: expected; AUC: area under curve; 95% CI: 95% confidence intervals; H-L: Hosmer–Lemeshow.

**Table 3:** Unadjusted and adjusted odds ratios for 24 h troponin T (TnT).

	Odds Ratio	95% Confidence Intervals
Unadjusted TnT at 24 h postoperatively	0.88	0.82–0.93
Adjusted * TnT at 24 h postoperatively	0.87	0.77–0.97

* We adjusted troponin T (TnT) for age, preoperative hematocrit, and Surgical Outcome Risk Tool (SORT) stratification.

**Table 4:** Patient demographics stratified by the normal or high levels of troponin T (TnT) preoperatively and at 24 and 48 h postoperatively.

Demographics	Pre-op TnT		TnT 24 h		TnT 48 h	
	Normal n = 189	High n = 6	Normal n = 166	High n = 29	Normal n = 167	High n = 27
Female, n (%)	48 (25.4)	1 (16.7)	42 (25.3)	7 (24.1)	42 (25.2)	7 (25.9)
Age ≥ 70 (%)	58 (30.7)	2 (33.3)	45 (27.1)	15 (51.7)	45 (26.9)	14 (51.9)
ASA Class, n (%)						
I	38 (20.1)	0 (0)	36 (21.7)	2 (6.9)	36 (21.6)	2 (7.4)
II	97 (51.3)	1 (16.7)	90 (54.2)	8 (27.6)	90 (53.9)	8 (29.6)
III	41 (21.7)	3 (50)	32 (19.3)	12 (41.4)	32 (19.2)	12 (44.4)
IV	13 (6.9)	2 (33.3)	8 (4.8)	7 (24.1)	8 (4.8)	6 (22.2)
SORT (SD)	1.3 (1.7)	4.5 (4.3)	1.1 (1.3)	2.7 (3.6)	1.2 (1.5)	2.7 (3.4)
Operative priority, n (%)						
Elective	156 (82.5)	2 (33.3)	142 (85.5)	16 (55.2)	142 (85.0)	16 (59.3)
Acute	33 (17.5)	4 (66.6)	24 (14.5)	13 (44.8)	24 (14.4)	12 (44.4)
Cancer site, n (%)						
Pancreas	161 (85.2)	3 (50)	144 (86.7)	20 (69)	144 (86.2)	19 (70.4)
Stage						
Resectable	118 (73.3)	2 (33.3)	103 (62.0)	7 (24.1)	103 (61.7)	7 (25.9)
Borderline resectable	53 (32.9)	1 (16.7)	41 (24.7)	13 (44.8)	41 (24.6)	12 (44.4)
PDAC	136 (84.5)	3 (50)	121 (72.9)	18 (62)	121 (72.5)	17 (63.0)
NET	14 (8.7)	0 (0)	12 (7.2)	2 (6.9)	12 (7.2)	2 (7.4)
Other	11 (6.8)	0 (0)	11 (6.6)	0 (0)	11 (6.6)	0 (0)
Hepatobiliary	28 (14.8)	3 (50)	22	9	22	9
Stage						
I, II	23 (82.1)	2 (66.7)	21 (12.7)	4 (13.8)	21 (12.6)	4 (14.8)
III	5 (17.9)	1 (33.3)	1 (0.6)	5 (17.2)	1 (0.6)	5 (18.5)
Neoadjuvant treatment, n (%)	85 (45.0)	2 (66.7)	73 (44.0)	14 (48.3)	73 (43.7)	14 (51.9)
Surgical operation, n (%)						
Pancreaticoduodenectomy	80 (42.4)	3 (50)	66 (39.8)	17 (58.6)	66 (39.5)	16 (59.3)
Distal pancreatectomy	12 (6.3)	0 (0)	10 (6.0)	2 (6.9)	10 (6.0)	2 (7.4)
Hepatectomy	29 (15.3)	3 (50)	23 (13.9)	9 (31.0)	23 (13.8)	9 (33.3)
Other procedures	68 (36.0)	0 (0)	67 (40.4)	1 (3.4)	67 (40.1)	1 (3.7)
Blood loss						
<100 mL	61 (32.3)	0 (0)	60 (36.1)	1 (3.4)	60 (35.9)	1 (3.7)
101–500 mL	89 (47.1)	3 (50)	78 (47.0)	14 (48.3)	78 (46.7)	14 (51.9)
501–1000 mL	35 (18.5)	1 (16.7)	25 (15.1)	11 (37.9)	25 (15.0)	11 (40.7)
>1001 mL	7 (3.7)	2 (33.3)	6 (3.6)	3 (10.3)	6 (3.6)	2 (7.4)
Anesthesia events, n (%)	6 (3.2)	3 (50)	5 (3.0)	4 (13.8)	5 (3.0)	3 (11.1)
Severity of procedure, n (%)						
Major/Complex	178 (94.2)	4 (66.6)	158 (95.2)	24 (82.8)	158 (94.6)	23 (85.2)
30-day mortality	11 (5.8)	2 (33.3)	3 (1.8)	10 (34.5)	3 (1.8)	9 (33.3)

Abbreviations: ASA: American Society of Anesthesiologists.

## Data Availability

Data is available upon request.
